# Health-Related Fitness as a Predictor of Anxiety Levels Among School Adolescents: An observational cross-sectional study

**DOI:** 10.2174/17450179-v18-e2208151

**Published:** 2022-10-13

**Authors:** Sandro Legey, Alberto Souza Sá Filho, Ali Yadollahpour, Fabio Garcia-Garcia, Claudio Imperatori, Eric Murillo-Rodriguez, Antonio Egidio Nardi, João Lucas Lima, Sergio Machado

**Affiliations:** 1 Laboratory of Panic and Respiration, Institute of Psychiatry, Federal University of Rio de Janeiro;, Rio de Janeiro, Brazil; 2 Post Graduate Program of University Center of Anápolis (UniEVANGÉLICA), Anápolis, Brazil;; 3 Department of Psychology, University of Sheffield, Sheffield, United Kingdom;; 4 Biomedicine Department, Health Science Institute, Veracruzana University, Xalapa, Veracruz, Mexico;; 5 Department of Human Sciences, European University of Rome, Rome, Italy;; 6 Laboratorio De Neurociencias Moleculares E Integrativas, Escuela De Medicina, División Ciencias De La Salud, Universidad Anáhuac Mayab, Mérida, Mexico;; 7 Laboratory of Physical Activity Neuroscience, Neurodiversity Institute, Queimados- RJ , Brazil;; 8 Department of Sports Methods and Techniques, Federal University of Santa Maria, Santa Maria, Brazil;; 9 Intercontinental Neuroscience Research Group, Mérida, México.

**Keywords:** Adolescents, Anxiety, Epidemiology, Fitness, Mental health, Physical activity

## Abstract

**Background:**

There is an inverse association between cardiorespiratory fitness and general anxiety levels in adolescents. Obesity also is associated with a higher risk of anxiety in this population. However, little is known about the association between other health-related fitness elements with anxiety symptoms in this population. The authors explored the relationship between health-related fitness and anxiety symptoms in a large sample of Brazilian youth.

**Methods:**

This was an observational cross-sectional study with a sample comprised of 257 school adolescents, who were 136 girls (52.9%) and 121 boys (47.1%). The health-related fitness elements were evaluated by FitnessGram® test and anxiety levels by Multidimensional Anxiety Scale for Children - 39. Hierarchical regression analyses were used to determine the association between health-related fitness elements and anxiety symptoms in both sexes.

**Results:**

In male adolescents, only the cardiorespiratory fitness was significantly associated with anxiety symptoms (*F*_(1, 119)_ = 6.472; *P* = 0.012; *R*^2^ = 0.052; adjusted *R*^2^ = 0.044). In turn, the anxiety symptoms showed an inverse small relationship with cardiorespiratory fitness (*r* = - 0.227; *P* < 0.01). However, in female adolescents, no association was found between health-related fitness elements and anxiety symptoms.

**Conclusion:**

The level of cardiorespiratory fitness may represent a marker of anxiety in male adolescents.

## 1 INTRODUCTION

The literature on mental disorders affecting children and adolescents showed that the worldwide prevalence of any anxiety disorder was 6.5% [1]. When present in childhood, these disorders are associated with other mental disorders, such as major depressive disorder [2], increased risk of suicide attempts [3], bipolar disorder, and schizophrenia [4, 5]. Moreover, due to adverse long-term psychopathological outcomes, child and adolescent anxiety disorders were linked to poor long-term functioning and general health, and interpersonal, and educational difficulties [6]. The early anxiety manifestations during infancy are at the outset of a “cascade of psychopathology,” where the course of pediatric anxiety disorders is overall considered to be chronic and persistent [7]. Thus, the early recognition of anxiety manifestations and the proper treatment are important.

There are complex predictors associated with anxiety disorders in adolescents such as age, sex, genetics, temperament, parenting behavior, environmental triggers, mother history of psychiatric hospitalization, mother education, residence, and physiologic factor [5, 8]. In addition, the presence of a medical comorbid also increases the odds of having a comorbid anxiety disorder [9]. Furthermore, the level of physical activity (PA) needs to be considered in mental health in adolescents [10-12]. PA is defined as any bodily movement produced by the contraction of skeletal muscles, resulting in energy expenditure above the energy expenditure at rest [13-16].A previous review showed that physical activity has small beneficial effects for reduced anxiety in adolescents [17]. Nevertheless, in an updated review, the authors suggested that due to the small number and diversity of studies and populations, a complete analysis of the association between physical activity and anxiety in children and adolescents still is considered premature [18].

Given that, health-related fitness elements have also been investigated concerning anxiety levels in children and adolescents. According to the American College of Sports Medicine (ACSM), the health-related fitness elements are cardiorespiratory endurance, body composition, muscular strength or endurance, and flexibility [13]. In this sense, higher levels of cardiorespiratory fitness (CRF) were associated with lower general anxiety levels in adolescents (≈18 years old) [19]. In addition, female adolescents (12-13 years old) who had better CRF levels were less susceptible to sleep-related anxiety problems [20]. About body composition, a study of 12,507 Swedish children and adolescents (6-17 years old) showed that obesity per se is associated with a higher risk of anxiety in this population [21]. In turn, the association of muscle strength and flexibility with anxiety levels in adolescents remains scarce in the literature. Therefore, knowledge of health-related fitness elements in this population would have important implications for symptoms of anxiety.

An important alternative to assess the health-related fitness elements would be through the FitnessGram® test [22]. The FitnessGram® has been used to evaluate four health-related fitness components commonly used by school adolescents [23] and provided teachers with practical tools to enhance physical education programming [24]. The method showed good accuracy and predictive ability when administered by trained teachers [25]. In addition, FitnessGram® is easy to apply and may represent an advancing practice of physical education in schools [26]. Due to the coronavirus disease 2019 (COVID-19), in large parts of the world, children and adolescents had their schools closed, government-mandated activity restrictions imposed, and interactions outside the home reduced. These restrictions had a considerable impact on PA levels and psychological status in adolescents [27]. Given this scenario, new tools must assess health-related fitness in school adolescents, especially during the pandemic. Thus, the present study aimed to examine the relationship between health-related fitness and anxiety symptoms in a large sample of Brazilian youth. We hypothesized that higher obesity levels would be associated with more severe anxiety symptoms, and higher levels of CRF with lower anxiety symptoms.

## 2 MATERIALS AND METHODS

### 2.1 Study Design

This was an observational cross-sectional study with 257 school adolescents aged between 13 and 19 years old. Some public schools in Búzios city, localized in Rio de Janeiro state, Brazil, were invited to participate in the study. Based on the schools' interest and feasibility, a total of two schools permitted study recruitment in one or several of their classrooms.

### 2.2 Setting

The experimental procedures were conducted during a regularly scheduled class time under the supervision of the research staff. The testing methods were conducted in four visits, with a week between them, during a regularly scheduled class time under the supervision of the research staff. On the first visit, students and parents were informed about all procedures and objectives of the study. After that, the signed informed consent was obtained from the students and their parents. In addition, the first visit was used to familiarize adolescents with the Multidimensional Anxiety Scale for Children - 39 (MASC-39) [28] and FitnessGram test [22]. In the second and third visits, the adolescents underwent a battery of fitnessgram tests. In the fourth visit, the adolescents responded only to MASC-39. The MASC-39 was applied to adolescents in the classroom with ambient temperature adjusted to 22° C. In turn, the FitnessGram test was performed in a sports gym.

### 2.3 Participants

In both schools, the students were enrolled from the fifth year of elementary school to the third year of medium school. Parental informed consent was obtained from 352 students enrolled in these schools. Any student with an individual education plan as a result of disability was excluded from the study. Moreover, any student who did not complete all FitnessGram tests or MASC-39 was excluded from the study. In this sense, 95 adolescents refused to participate in the FitnessGram^®^ test. Thus, the study sample comprised 257 adolescents, 70.6% of those initially recruited. Of the total number were 121 boys and 136 girls, respectively. The institutional ethics committee obtained ethics approval for this study (Pro 3.105.242).

### 2.4 Variables

The assessment of health-related fitness was performed by FitnessGram tests [22]. The FitnessGram^®^ was developed to increase parental awareness of physical fitness assessments in children and adolescents [22]. The FitnessGram test is a valid and reliable [25] battery of assessments used to identify the four health-related fitness components. The fitness tests were completed in three visits during regularly scheduled physical education classes and administered by a trained research team that provided standardized encouragement for participants during all test phases. During the first visit, they were familiarized with each test through demonstration and were allowed to practice the testing protocols. During the second visit, the adolescents underwent a body composition assessment and, in the following, completed the Progressive Aerobic Cardiovascular Endurance Run (PACER). During the third visit, the back saver, sit and reach test and curl-up test was assessed, with an interval of 10 minutes between the tests.

Body composition was estimated by bioelectrical impedance analysis (BIA) (Omron^®^, model HBF-306, USA). In addition, height and weight were measured (Filizola model 31, Filizola S.A., São Paulo, Brazil) and converted to body mass index (BMI). Anthropometric measurements were performed according to the ACSM technical procedures [13]. To perform the evaluation using the BIA technique, the adolescents received the following previous instructions: (1) not to take diuretic medications in the last seven days; (2) be fasting for at least 4 hours; (3) not drink alcoholic beverages in the last 48 hours; (4) not engaging in intense physical activity in the last 24 hours; (5) urinating at least 30 minutes before the evaluation; (6) removing objects such as earrings, bracelets, necklaces and piercings from the body.

The PACER is used to assess CRF in a 20-m shuttle run that progressively increases in difficulty. This test involves running back and forth across a 20-meter course in time determined by beeps on the soundtrack to indicate when a person should reach the period end. The test begins at a slow pace, and each minute the pace increases until the speed can no longer be maintained. The rate of estimated oxygen uptake (VO_2max_) is predicted from the number of laps completed during the test [29]. The data from the PACER test were processed using both the Leger equation [30] and the new test equating procedure [29].

The back saver, sit and reach test was performed for assessment of lower body flexibility [22]. The adolescent seated on the mat kept a lower limb extended with his foot resting on the well's bench. The other lower limb remained with the flexed knees and foot on the mat and next to the knee of the other member. The adolescent then flexed the trunk four times forward so that the hands slid over the measurement scale on the Well bench and remained in their maximum range of motion so that the evaluator could annotate the distance reached. These trunk flexion movements were alternated between the limbs, with the highest value achieved by each limb being considered, generally in the fourth flexion. The mean between the highest values obtained was noted [22].

The curl-up test was performed for the assessment of abdominal muscle endurance [22]. The adolescent laid supine, with knees bent and feet resting on the mat. A strip of 11.4 cm in width and 80 cm in length was placed on the mat under the adolescent’s knees, while the adolescent kept the arms extended at the side of the body and touched the strip with the fingertips. The exercise consisted of flexing the trunk until the fingers of the hands touched the other side of the strip, in a constant rhythm of repetition (one every three seconds). The number of repetitions performed correctly was noted [22].

The Multidimensional Anxiety Scale for Children - 39 (MASC-39) assessed anxiety symptoms in student adolescents. The MASC-39 is a 39-item, 4-point Likert-style self-report scale that evaluates anxiety symptoms across the four basic anxiety dimensions (physical symptoms, harm avoidance, social anxiety, and separation anxiety/panic) [28]. The MASC-39 has demonstrated satisfactory internal reliability and excellent stability in children and adolescents [28]. In addition, the MASC-39 has been translated to Portuguese and validated for use in children and adolescents [31]. For example, the Brazilian study by Vianna [31] determined in children and adolescents in Rio de Janeiro that ≥ 56 points were considered symptomatic concerning the general state of anxiety.

### 2.5 Statistical Analysis

Adolescents' characteristics were described in both sexes. The Chi-square test was used to analyze the association between anxiety status and sex. All variables were examined for outliers, normality, and collinearity among continuous explanatory variables, and all assumptions for hierarchical linear regression were met. The Pearson correlation was used to analyze the association between variables of interest. The correlation coefficients were interpreted using the scale of magnitudes proposed by Hopkins (www.sportsci.org): < 0.1, trivial; 0.1-0.29, small; 0.3-0.49, moderate; 0.5-0.69, large; 0.7-0.89, very large; > 0.9, nearly perfect. The hierarchical regression was done considering the hierarchical nature of the relationship among the predictor variables, as shown in the introduction and Pearson's correlation analysis. It has been suggested that cardiorespiratory fitness [19] and obesity [21] were related to anxiety in adolescents. Thus, four predictive models of anxiety levels in boys and girls were constructed: In the first model, we included cardiorespiratory fitness (Block 1). Secondarily, we add the percentage of body fat for the second model (Block 2). Then, in the third model, we included muscle endurance (Block 3). Finally, we add flexibility for the fourth model (Block 4). Analyses were conducted using the statistical package for the social sciences (SPSS Inc., New York) version 21.0, with an alpha level of 0.05.

## 3 RESULTS

### 3.1 Descriptive Characteristics

Sample characteristics are presented in Table **1**. The sample was approximately evenly comprised of males (47.1%) and females (52.9%) of 257 adolescents in total. Mean anxiety scores were below the clinical cut-off values generally used for anxiety (≥ 56) in males and females. In addition, the health-related fitness elements in male and female adolescents are presented in Table **1**.

Regarding the state of anxiety, in a total of 136 female adolescents, 43 were symptomatic (31.6%), and 93 did not show anxiety symptoms (68.4%). Regarding male adolescents, 20 were symptomatic (16.5%), and 101 did not show anxiety symptoms (83.5%). There was a significant association between sex and anxiety status (*X*^2^ (1) = 7.878; *Phi* = 0.175; *P* = 0.005). Female adolescents were 2.36 times more at risk to present anxiety symptoms than male adolescents (95% CI = 1.281, 4,257).

### 3.2 Correlations

Bivariate correlations between variables of interest are shown in Table **2**. In turn, in boys, the anxiety symptoms led to a small inverse relationship with cardiorespiratory fitness (*r* = - 0.227). Moreover, a small correlation was found between anxiety symptoms and the percentage of body fat in males (*r* = 0.214). In turn, in female adolescents, the relationship between health-related fitness elements with anxiety symptoms was only trivial.

### 3.3 Hierarchical Regression Analysis

Results of the regression predicting symptoms of anxiety are presented in Table **3**. In male adolescents, only the first model (Block 1) was significantly associated with severity of anxiety symptoms (*P* = 0.012; *R*^2^ = 0.052; adjusted *R*^2^ = 0.044). However, no association was found for the model 2 (*P* = 0.140; *R*^2^ = 0.069; adjusted *R*^2^ = 0.053), model 3 (*P* = 0.855; *R*^2^ = 0.069; adjusted *R*^2^ = 0.045), or model 4 (*P* = 0.303; *R*^2^ = 0.078; adjusted *R*^2^ = 0.046) with of anxiety symptoms in male adolescents. Thus, the symptoms of anxiety were significantly associated with cardiorespiratory fitness (Table **3**).

However, in female adolescents, no association was shown between the model 1 (*P* = 0.136; *R*^2^ = 0.016; adjusted *R*^2^ = 0.009), model 2 (*P* = 0.767; *R*^2^ = 0.017; adjusted *R*^2^ = 0.002), model 3 (*P* = 0.157; *R*^2^ = 0.032; adjusted *R*^2^ = 0.010), or model 4 (*P* = 0.536; *R*^2^ = 0.035; adjusted *R*^2^ = 0.005) with of anxiety symptoms. Results of the regression predicting symptoms of anxiety in male and female adolescents are presented in Table **3**.

## 4 DISCUSSION

The present study examined the relationship between health-related fitness and general anxiety levels in a large community sample of Brazilian adolescents. Results indicate that only the CRF showed a small inverse relationship with anxiety symptoms in male adolescents. However, no association was found between health-related fitness and anxiety symptoms in girls. In fact, this was the first study that explored the relationship between health-related fitness components and mental health in adolescents, although other studies have addressed this issue, the analysis of the present study is more complete, including more aspects related to health.

Furthermore, few studies have explored the relationship between CRF and anxiety state in adolescents [19, 32-34]. In turn, the increase in CRF has been related to lower levels of trait anxiety in healthy adults [35-37], mainly in patients with anxiety disorders. In this scenario, the anxiolytic effects of physical activity are well documented only in this population [38], which may limit an explanation of our findings. Our results suggest a small association between the lowest levels of cardiorespiratory fitness and the highest levels of general anxiety in male adolescents. Indirect evidence corroborates that the relationship between cardiorespiratory fitness and general anxiety state can be influenced by sex [39]. For instance, lower CRF in boys increased the likelihood of nervousness by 1.24 times. In female adolescents, lower CRF increased the likelihood of irritability by 1.17 times [39]. However, contrary to this hypothesis, Williams *et al*. [19] demonstrated that higher levels of CRF were associated with lower levels of general anxiety, where a large part of the sample was composed of female adolescents (81%). Because of these disagreements, Williams *et al*. [19] proposed the potential mechanism explaining the cardiorespiratory fitness-anxiety relationship. They suggested that higher levels of CRF were positively associated with more positive perceptions of anxiety symptoms and lower levels of state anxiety [19]. Corroborating this hypothesis, Cadenas-Sanchez *et al*. [40] showed that a higher CRF level was associated with higher motivation and lower anxiety, especially in female adolescents. In this sense, the available information to date only suggests that improvements in CRF positively affect general anxiety in young people. In terms of practical application, it seems essential to include more motivational activities in physical education lessons to increase adolescents’ CRF levels and possibly reduce general levels of anxiety.

Furthermore, our findings also found no relationship between other health-related fitness components (*e.g*., body composition, muscle endurance, and flexibility) and anxiety symptoms in male and female adolescents. Although physical fitness positively affects skeletal health during childhood and adolescence, the association between physical fitness and mental health in young people is still scarce [33]. Regarding the body composition of adolescents, the literature has been consistent in demonstrating that the prevalence of anxiety symptoms among overweight/obese adolescents is higher than that in normal-weight adolescents [21, 41, 42]. Previous research has shown bidirectional associations between obesity and anxiety in adolescents [41, 42]. In turn, obesity per se is associated with a high risk of anxiety in adolescents [21]. Despite the small relationship found between the percentage of fat and anxiety levels in male adolescents (Table **2**), our results found no showed predictor effect of body fat percentage on anxiety levels in this sample (Table **3**). In addition, no relationship has been demonstrated between the percentage of body fat and anxiety levels in male adolescents. In part, this can be explained by the values obtained for the percentage of body fat in both groups (Table **1**), which are in accordance with the age and sex criteria established by the Cooper Institute of Dallas as healthy fitness zones that offer protection against diseases that result from sedentary [22].

Regarding the relationship between muscle endurance and flexibility with general levels of anxiety, data in the literature are scarce. Specifically, in adolescents, a previous study [43] showed a higher mean decrease in anxiety levels in the intervention group (pain neuroscience education plus muscle endurance for neck flexor and extensors) than in the control group, but the differences did not reach statistical significance. In addition, no relationship was established between muscle endurance and general anxiety levels in adolescents. To our knowledge, there are no studies that have explored the relationship between flexibility and general levels of anxiety.

### 5 LIMITATIONS

Some limitations of the study should be noted. First, the study design was based on a cross-sectional survey and, consequently, causation cannot be determined. Second, the FitnessGram test assesses health-related fitness instead of the gold standard procedure. However, in terms of population data and the high costs for implementation and time spent on testing, the FitnessGram tests may represent an advancing physical education practice in schools [26]. Finally, the anxiety scale was translated and validated by a master's research [31]. Although our sample is similar to that used by Vianna [31], his research did not receive blind peer evaluation and independently from any scientific journal.

## CONCLUSION

In conclusion, only the cardiorespiratory fitness of boys showed a small inverse association with the state of anxiety. Thus, the level of cardiorespiratory fitness may represent a marker of anxiety in boys. This is an important finding because the FitnessGram test may be easily used to assess health-related fitness components in adolescents. In addition, this test made it possible to identify a small relationship between CRF and mental health status.

## Figures and Tables

**Table 1:** Sample characteristics.

**Boys (n = 121)**	**M**	**SD**	**95% CI**
Age (years)	15.6	1.1	15.4 – 15.8
Stature (m)	1.7	0.1	1.7 – 1.7
Body mass (kg)	62.5	12.6	60.2 – 64.7
Body mass index (kg/m^2^)	21.2	4.5	20.5 – 21.9
% BF	18.5	8.0	17.0 – 19.9
CRF (mL.kg^-1^.min^-1^)	32.9	7.8	31.5 – 34.3
ME (reps)	35.0	20.8	31.2 – 38.7
Flexibility (cm)	26.8	7.8	25.4 – 28.2
Anxiety (MASC-39 total score)	36.5	16.7	33.5 – 39.5
**Girls (n = 136)**	**M**	**SD**	**95% CI**
Age (years)	15.3	1.0	15.2 – 15.5
Stature (m)	1.6	0.1	1.6 – 1.6
Body mass (kg)	57.3	12.5	55.2 – 59.4
Body mass index (kg/m^2^)	22.0	4.1	21.3 – 22.7
% BF	25.2	6.6	24.1 – 26.3
CRF (mL.kg^-1^.min^-1^)	23.3	4.6	22.6 – 24.1
ME (reps)	25.5	19.2	22.2 – 28.7
Flexibility (cm)	29.9	7.0	28.7 – 31.1
Anxiety (MASC-39 total score)	48.1	16.0	45.4 – 50.8

% BF: percentage of body fat; CRF: cardiorespiratory fitness; ME: muscle endurance; m: meters; kg: kilogram, mL.kg^-1^.min^-1^: milliliters of oxygen per kilogram of body weight per minute; reps: repetitions; cm: centimeter; MASC-39: Multidimensional Anxiety Scale for Children – 39; M: mean; SD: standard deviation; 95% CI: 95% Confidence Intervals.

**Table 2:** Correlations between variables of interest.

Boys	CRF	% BF	ME	Flexibility	Anxiety
CRF	-	- 0.411^#^	0.272**	0.186*	- 0.227**
% BF	- 0.411^#^	-	- 0.177*	- 0.134	0.214**
ME	0.272**	- 0.177*	-	0.238**	- 0.056
Flexibility	0.186*	- 0.134	0.238**	-	0.041
Anxiety	- 0.227**	0.214**	- 0.056	0.041	-
Girls	CRF	% BF	ME	Flexibility	Anxiety
CRF	-	- 0.409^#^	0.246**	0.074	- 0.128
% BF	- 0.409^#^	-	- 0.090	- 0.016	0.029
ME	0.246**	- 0.090	-	0.218**	0.086
Flexibility	0.074	- 0.016	0.218**	-	0.067
Anxiety	- 0.128	0.029	0.086	0.067	-

CRF: cardiorespiratory fitness; % BF: percentage of body fat; ME: muscle endurance.

*P < 0.05, two-tailed; **P < 0.01, two-tailed; #*P* ≤ 0.0001, two-tailed

**Table 3:** Hierarchical linear regression analysis of predictors of anxiety.

** Model **	**Boys**	** B **	**SE**	** β **	** t **	** P **	**95% CI**	**Tolerance**	**VIF**
Block 1	CRF	- 0.486	0.191	- 0.227	- 2.544	0.012	- 0.865, - 0.108	1.000	1.000
Block 2	CRF	- 0.359	0.209	- 0.168	- 1.721	0.088	- 0.772, 0.054	0.831	1.203
	% BF	0.303	0.204	0.145	1.485	0.140	- 0.101, 0.707	0.831	1.203
Block 3	CRF	- 0.368	0.215	- 0.172	- 1.711	0.090	- 0.794, 0.058	0.790	1.266
	% BF	0.306	0.206	0.146	1.489	0.139	- 0.101, 0.713	0.827	1.210
	ME	0.014	0.075	0.017	0.184	0.855	- 0.134, 0.162	0.921	1.086
Block 4	CRF	- 0.391	0.216	- 0.182	- 1.808	0.073	- 0.818, 0.037	0.782	1.279
	% BF	0.317	0.206	0.151	1.539	0.126	- 0.091, 0.724	0.824	1.213
	ME	- 0.002	0.076	- 0.002	- 0. 020	0.984	- 0.152, 0.149	0.886	1.129
	Flexibility	0.204	0.198	0.096	1.034	0.303	- 0.187, 0.596	0.925	1.081
** Model **	**Girls**	** B **	**SE**	** β **	** t **	** P **	**95% CI**	**Tolerance**	**VIF**
Block 1	CRF	- 0.448	0.299	- 0.128	- 1.499	0.136	- 1.040, 0.143	1.000	1.000
Block 2	CRF	- 0.488	0.322	- 0.140	- 1.484	0.140	- 1.139, 0.162	0.833	1.201
	% BF	- 0.068	0.230	- 0.028	- 0.296	0.767	- 0.524, 0.387	0.833	1.201
Block3	CRF	- 0.598	0.337	- 0.171	- 1.777	0.078	- 1.264, 0.068	0.789	1.268
	% BF	- 0.072	0.229	- 0.029	- 0.313	0.754	- 0.526, 0.382	0.833	1.201
	ME	0.105	0.074	0.126	1.425	0.157	- 0.041, 0.251	0.940	1.064
Block 4	CRF	- 0.603	0.337	- 0.173	- 1.788	0.076	- 1.271, 0.064	0.788	1.268
	% BF	- 0.074	0.230	- 0.030	- 0.321	0.749	- 0.529, 0.381	0.833	1.201
	ME	0.095	0.076	0.114	1.263	0.209	- 0.054, 0.245	0.900	1.112
	Flexibility	0.125	0.201	0.055	0.620	0.536	- 0.273, 0.522	0.952	1.050

CRF: cardiorespiratory fitness; % BF: percentage of body fat; ME: muscle endurance

## LIST OF ABBREVIATIONS

ACSM: = American College of Sports Medicine
CRF: = Cardiorespiratory Fitness
COVID-19: = coronavirus disease 2019
PACER: = Progressive Aerobic Cardiovascular Endurance Run
